# The conservation and uniqueness of the caspase family in the basal chordate, amphioxus

**DOI:** 10.1186/1741-7007-9-60

**Published:** 2011-09-21

**Authors:** Liqun Xu, Shaochun Yuan, Jun Li, Jie Ruan, Shengfeng Huang, Manyi Yang, Huiqing Huang, Shangwu Chen, Zhenghua Ren, Anlong Xu

**Affiliations:** 1 State Key Laboratory of Biocontrol, National Engineering Research Center of South China Sea Marine Biotechnology, Department of Biochemistry, College of Life Sciences, Sun Yat-sen University, 135 West Xingang Road, Guangzhou, 510275, PR China

## Abstract

**Background:**

The caspase family, which plays a central role in apoptosis in metazoans, has undergone an expansion in amphioxus, increasing to 45 members through domain recombination and shuffling.

**Results:**

In order to shed light on the conservation and uniqueness of this family in amphioxus, we cloned three representative caspase genes, designated as *bbtCaspase-8, bbtCaspase-1/2 *and *bbtCaspase3*-like, from the amphioxus *Branchiostoma belcheri tsingtauense*. We found that *bbtCaspase-8 *with conserved protein architecture is involved in the Fas-associated death domain-Caspase-8 mediated pro-apoptotic extrinsic pathway, while *bbtCaspase3*-like may mediate a nuclear apoptotic pathway in amphioxus. Also, *bbtCaspase-1/2 *can co-localize with *bbtFADD2 *in the nucleus, and be recruited to the cytoplasm by amphioxus apoptosis associated speck-like proteins containing a caspase recruitment domain, indicating that *bbtCaspase-1/2 *may serve as a switch between apoptosis and caspase-dependent innate immune response in invertebrates. Finally, amphioxus extrinsic apoptotic pathway related caspases played important roles in early embryogenesis.

**Conclusions:**

Our study not only demonstrates the conservation of *bbtCaspase-8 *in apoptosis, but also reveals the unique features of several amphioxus caspases with novel domain architectures arose some 500 million years ago.

## Background

Programmed cell death is a gene-guided process for the elimination of unnecessary or harmful cells in which the cysteine proteases caspases are core elements [1-3]. To date, 11 members of this family have been identified in the human genome [4,5], caspase-1 to caspase-10, and caspase-14. They can be divided, with respect to function, into two major groups. The first, including the initiator caspases (caspase-2, -8, -9 and -10) and the effector caspases (caspase-3, -6 and -7), lead directly to apoptosis [6], while the others, including caspase-1, -4 and -5, are involved in the maturation of pro-inflammatory cytokines [7]. Caspase-14 is mainly expressed in the epidermis and takes part in epidermal barrier formation [8].

In vertebrate cells, the apoptotic response is mediated through either the intrinsic or extrinsic pathway. The intrinsic pathway is triggered by death stimuli generated within the cell, such as DNA damage, leading to the release of mitochondrial cytochrome c, which associates with caspase-9 and apoptotic protease activating factor 1 (Apaf-1) to form an apoptosome [9]. The extrinsic apoptotic pathway is initiated by the binding of extracellular death ligands to death receptors (DRs), such as FasL binding to Fas. As members of the TNF receptor (TNFR) superfamily, the DRs further recruit the cytosolic adaptor Fas-associated death domain (FADD), which interacts with procaspase-8 through death effector domains (DEDs) to form an oligomeric death-inducing signaling complex [10]. The activated caspase-8 released subsequently cleaves the effector caspase-3, which finally executes apoptosis [11]. In addition to apoptosis, caspase-8 and caspase-3 are also involved in non-apoptotic functions. In the development of the yolk sac vasculature during mouse embryogenesis, caspase-8 restricts the RIP3-dependent pathway rather than apoptosis [12], and caspase-3 is required for the differentiation of embryonic stem cells and hematopoietic stem cells [13,14].

Another major cellular program mediated by caspases is cytokine processing [15]. In mammals, these caspases are distinctive due to having a caspase recruitment domain (CARD) at the N-terminal. Caspase-1 is the most documented of these. Caspase-1 activity is regulated by the nucleotide olimerization domain-like receptor (NLR) family members through inflammasome formation. These multiprotein complex formations usually require the adaptor apoptosis-associated speck-like protein (ASC), which contains an N-terminal pyrin domain (PYD) interacting with that of the NLR along with a C-terminal CARD with caspase-1. The activated caspase-1 controls maturation of interleukins such as IL-1β and IL-18, which direct a wide variety of effects related to innate immunity and host responses [16].

Homologs of the three proteins, CED-9, CED-4, and CED-3 (Bcl-2, Apaf-1 and caspase in mammals), which were first identified as being involved in apoptosis in nematodes, are found in the genomes of all animals and associated with apoptosis signaling [17]. It is logical to expect that an apoptotic regulatory network composed of Bcl-2, Apaf-1 and caspase had already been established in protostomes and was conserved to evolve into the intrinsic pathway in deuterostomes. However, cytochrome c binding has not been recognized for CED-4 in *Caenorhabditis elegans*, and *Drosophila *Apaf-1 homolog, Dark still remains controversial [18]. Moreover, although a dFADD and a TNFR have been described, such ancient TNFR lacks the death domain (DD) and does not recruit the dFADD [19], suggesting that the functional extrinsic apoptotic pathway does not exist in *Drosophila*. In addition, the inflammasome related caspases have been reported only in vertebrates. However, with the annotation of amphioxus immune related genes, an expanded caspase family has been identified in which many genes related to the extrinsic apoptosis pathway, and some novel genes related to inflammation, were identified. Thus, further functional analysis of the amphioxus caspase family will help to clarify whether the caspase-mediated extrinsic apoptosis and the caspase-dependent innate immune complex were completely formed, and what their unique features are, at the basal chordate stage.

## Results

### Analysis of three caspase genes with death-fold domains in amphioxus

**Figure 1:**
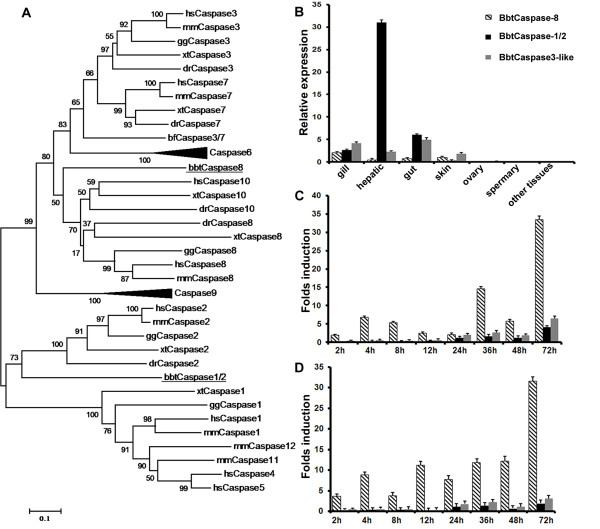
**Expression analysis of three caspase genes with death-fold domains in amphioxus**. **(A) **The two amphioxus initiator caspases and the caspase family of seven other organisms were used to construct a phylogenetic tree based on their caspase domains using MEGA version 3.1 (neighbor joining tree). Numbers at nodes indicate bootstrap values. hs: *Homo sapiens*; mm: *Mus musculus*; gg: *Gallus gallus*; xt: *Xenopus tropicalis*; dr: *Danio rerio*; sp: *Strongylocentrotus purpuratus*; bf: *Branchiostoma floridae*; bbt: *Branchiostoma belcheri tsingtauense*. **(B) **Quantitative RT-PCR was performed to determine tissue distribution of three caspases in adult amphioxus. Data are expressed as a ratio of the mRNA abundance as calculated with the 2^-ΔΔCt ^method from two parallel experiments performed in triplicate. **(C) **and **(D) **Quantitative RT-PCR analysis of three amphioxus caspases at 2, 4, 8, 12, 24, 36, 48, and 72 h after challenge with *S. aureus *(Gram-positive) and *V. vulnificus *(Gram-negative). Results are presented as the fold induction in the abundance of mRNA relative to that of samples exposed to PBS and were determined by the 2^-ΔΔCt ^method from two parallel experiments performed in triplicate. The endogenous control for normalization was mRNA for cytoplasmic β-actin. Values were considered to be significant when *P *< 0.05.

Our previous annotation reported that, due to the domain recombination and shuffling at the transition from invertebrates to vertebrates, the amphioxus genome contains at least 45 caspase genes [20], including 18 genes related to both caspase-9 and caspase-2, 15 to caspase-8/10, five to caspase-3/6/7, and seven to unknown caspase genes. Based on genomic analysis, from each of the known branches above we chose one caspase with a conserved domain and two with novel domain architecture for further analysis. These were *bbtCaspase-8*, which is the ortholog of caspase-8 with conserved protein architecture, *bbtCaspase-1/2*, which shares the greatest similarity with caspase-2, but its prodomain contains a DED and a DD instead of a CARD in mammalian caspase-2 (Figure 1A) and finally *bbtCaspase3*-like, which is 33% homologous to the human caspase-3/7 caspase domain (see Additional file 1).

To obtain clues to the functions of distinct caspases, we performed quantitative real-time PCR to detect their tissue distribution (Figure 1B) and responses to infection. The transcripts of all characterized caspases, especially *bbtCaspase-1/2*, were abundant in the hepatic cecum, gill and gut. Since the hepatic cecum and gut are considered as the first line of the amphioxus immune defense system, we proposed that the caspase family should play important roles in amphioxus immunity. In response to *Staphylococcus aureus *and *Vibrio vulnificus *challenges, the transcripts of *bbtCaspase-8*, but not of *bbtCaspase3*-like or *bbtCaspase-1/2*, were upregulated dramatically (Figures 1C and 1D). The distinct expression patterns of caspases may suggest their diverse functions in amphioxus immunity or other roles.

### Structure and function conservation of *bbtCaspase-8 *in apoptosis

**Figure 2:**
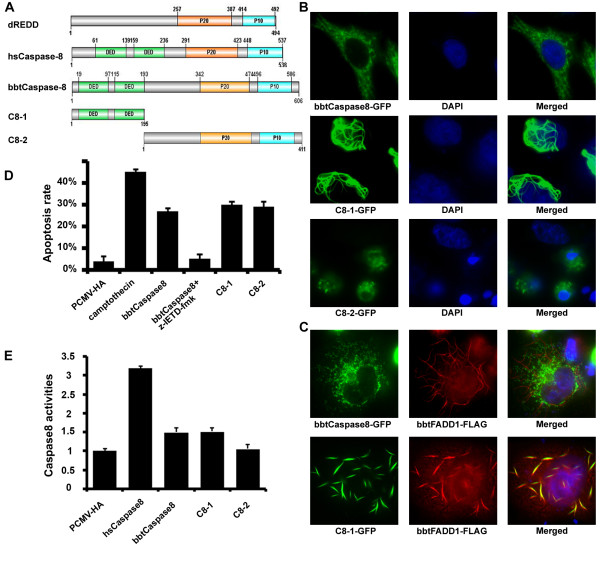
**BbtCaspase-8 interacted with DED of bbtFADD1 and induced HeLa cell apoptosis**. **(A) **Comparison of the domains of bbtCaspase-8 and its mutants with those of dDREDD and hsCaspase-8. **(B) **Subcellular localization of bbtCaspase-8-GFP and its mutants. **(C) **Overexpression of bbtFADD1 did not co-localize with full-length of bbtCaspase-8, but co-localized with prodomain mutant C8-1. **(D) **Annexin V PE staining was analyzed (excitation at 488 nm and emission at 578 nm) from cells transfected with indicated plasmids. The third bar represented the sample in the presence of 50 μM z-IETD-fmk. The negative control was transfected with vector pCMV and the positive control was treated with camptothecin at 10 μM for 24 h. Apoptosis rates were expressed as annexin V positive cells/10,000 gated cells. **(E) **Caspase-8 activity in HeLa cells was measured after 20 h transfection with indicated plasmids. Data from experiments with hsCaspase-8 was presented as a positive control. All data shown were means ± standard deviations of three samples for each treatment and values were considered to be significant when *P *< 0.05. Results were confirmed by at least three separate experiments.

*BbtCaspase-8 *exhibits similar protein structure to its vertebrate counterparts, including a pair of DEDs in the prodomain and a C-terminal caspase domain (Figure 2A). To compare its function to that of human caspase, full-length and several truncated versions of *bbtCaspase-8 *were fused with GFP for cell localization analyses. When expressed in HeLa cells, *bbtCaspase-8*-GFP protein was localized unevenly in the cytoplasm. The truncated mutant containing only the prodomain (C8-1) was shown to have death effector filamentous structures (DEF) [21] around the nucleus to promote cell death (Figures 2B and 2D). The prodomain (C8-1) of *bbtCaspase-8 *co-localizes with *bbtFADD1*, but not with *bbtFADD2 *(Figure 2C and Additional file 2). These results were in agreement with our previous report that, in HeLa cells, *bbtFADD1 *is expressed in the cytoplasm, while *bbtFADD2 *is restricted to the nucleus [22]. To determine if *bbtCaspase-8 *can induce apoptosis when overexpressed, we investigated the binding activity of *bbtCaspase-8 *transfected HeLa cells with annexin V, which is an early hallmark of apoptosis [23]. We found that full-length *bbtCaspase-8 *and two truncated mutants can induce apparent apoptosis, and that the apoptosis induced by full-length *bbtCaspase-8 *can be inhibited by the caspase-8 inhibitor z-IETD-fmk (Figure 2D). In addition, the caspase-8 activity in the transfected HeLa cell is 1.5 to 2-fold that of the negative control (Figure 2E). The sequence comparison of catalytic domain between *bbtCaspase-8 *and human caspase-8 indicated that these two amino acid sequences share more than 50% similarities (see Additional file 3). These results suggest that *bbtCaspase-8 *has substrate specificity similar to human caspase-8. Thus, we propose that *bbtCaspase-8 *can induce apoptosis through a direct DED-mediated interaction with *bbtFADD1 *[24] resulting in the cleavage of *bbtCaspase-8 *via the conservative catalytic site sequence QACQG.

### A DED-containing nuclear caspase can induce mammalian cell apoptosis

**Figure 3:**
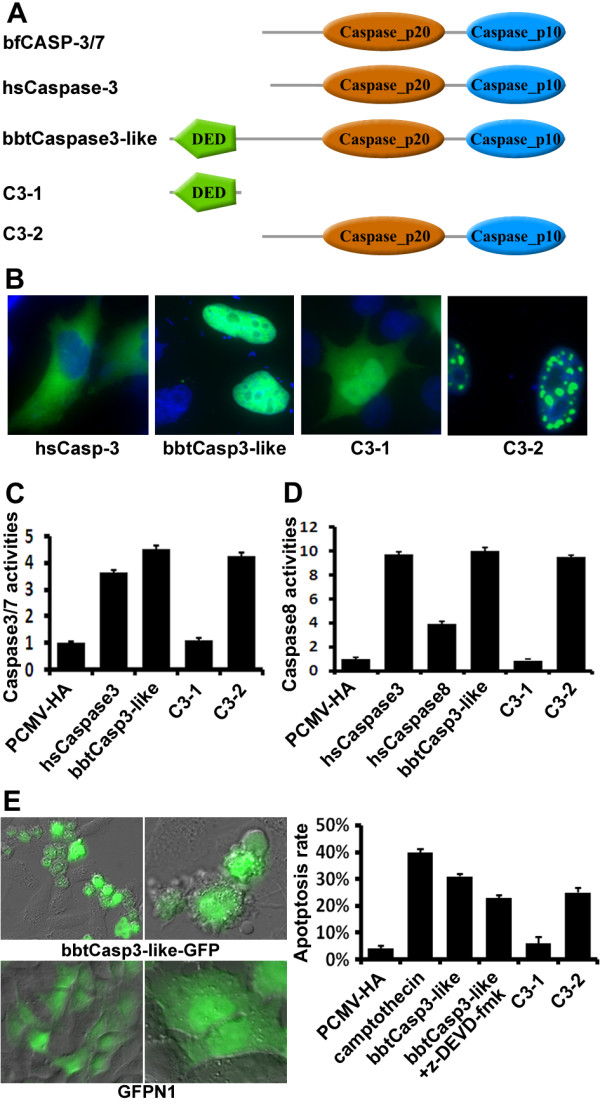
**Nuclear bbtCaspase3-like induced apoptosis independent of its DED**. **(A) **Comparison of domain structures among bfCASP-3/7, human caspase-3, bbtCaspase3-like, and its mutants. **(B) **BbtCaspase3-like-GFP and C3-2-GFP both localized in nucleus, while C3-1-GFP only containing DED localized ubiquitously in cells. These indicated that bbtCaspase3-like expressed in nucleus independent of its DED. **(C) **and **(D) **Caspase-3 and caspase-8 activity of HeLa cells tranfected with different plasmids. **(E) **The photograph and magnified view of HeLa cells displaying predominantly apoptotic morphology after transient transfection with plasmid encoding bbtCaspase3-like-GFP (20 ×, 63 × objectives). The morphology of cells transfected with GFPN1 vector was as the control. Apoptosis rates were expressed as annexin V positive cells/10,000 gated cells. Data were shown as percent control of the means ± standard deviation of three samples. The negative control was transfected with pCMV vectors and the positive control was treated with camptothecin at 10 μM for 24 h. The third bar represented the sample in the presence of 50 μM z-DEVD-fmk.

Compared to the initiator caspases and interleukin-converting enzyme (ICE) -like caspases, the *bbtCaspase3*-like caspase domain is more similar to the effector caspases, caspase-3 and caspase-7, in spite of its DED-containing prodomain that is not linked to the reported effector caspases (Figure 3A). Unlike mammalian caspase-3 and caspase-7, which are mainly present in cytoplasm [25], the fusion protein *bbtCaspase3*-like-GFP is expressed only in the nucleus of HeLa cells, and this localization is not required for the prodomain DED (Figure 3B). With a conserved active site, Cys330, within the catalytic motif QSCRG [11], the full length *bbtCaspase3*-like and the truncated mutant C3-2 lacking the prodomain were shown to promote cell death in transfected HeLa cells, in which increased caspase-3 and caspase-8 activities were also detected (Figures 3C and 3D). The apoptosis processes were further confirmed by visually observable apoptotic morphology and annexin V binding assays (Figure 3E). However, apoptosis was not completely inhibited by the caspase-3 inhibitor z-DEVD-fmk. Thus, compared to mammalian caspase-3 and -7, *bbtCaspase3*-like is a novel caspase with similar apoptotic activity but different protein architecture and cell localization.

### *BbtCaspase-1/2 *interacts with *bbtFADD2 *and translocates from the nucleus to the cytoplasm depending on *bbtASC*s

**Figure 4:**
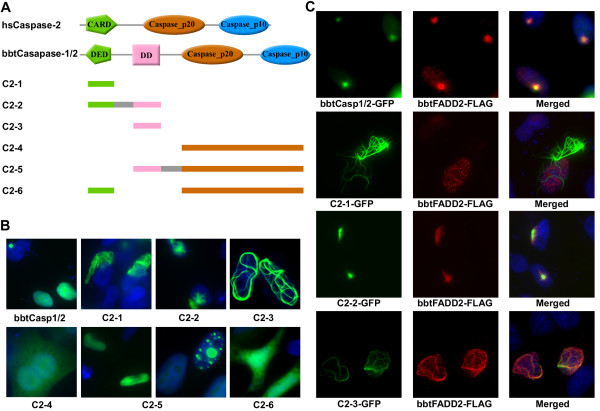
**BbtCaspase-1/2 co-localized with bbtFADD2 in nucleus**. **(A) **Comparison of the domains of bbtCaspase-1/2 with those of hsCaspase-2. Designations and structures of bbtCaspase-1/2 mutant fusion proteins used in this study. **(B) **Analysis of the subcelluar localization of bbtCaspase-1/2-GFP and several truncated mutants. **(C) **Overexpression of bbtCaspase-1/2 and the mutants containing the DD results in their co-localiztion with bbtFADD2 in the nucleus.

**Figure 5:**
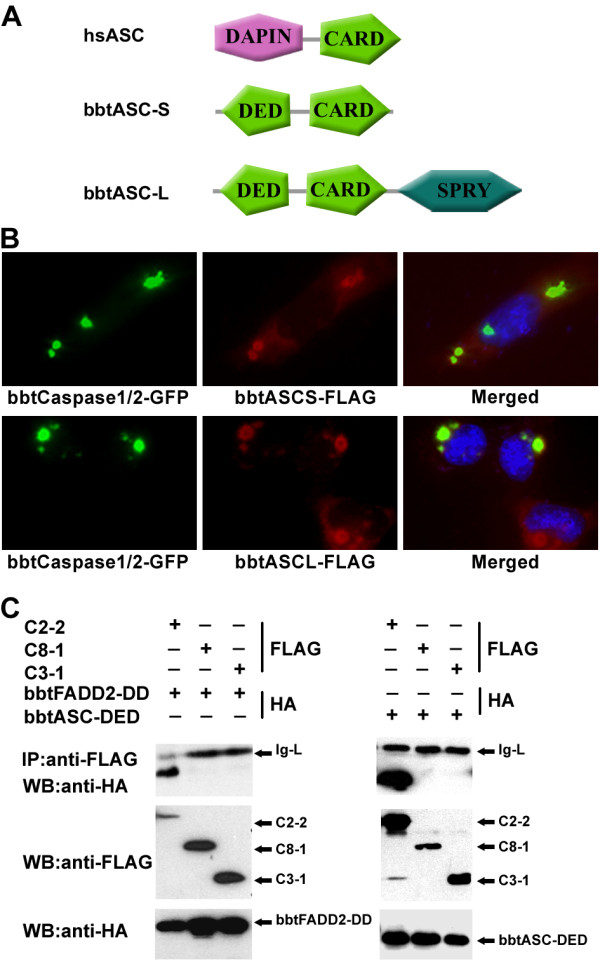
**BbtCaspase-1/2 translocated from nucleus to cytoplasm dependent on bbtASCs**. **(A) **Comparison of the domains of hsASC with those of the bbtASC-S and the bbtASC-L. **(B) **BbtASC-S and bbtASC-L both led to bbtCaspase-1/2 translocation from nucleus to cytoplasm and co-localization. **(C) **Co-immunoprecipitation experiments performed in HeLa cells confirmed that bbtCaspase-1/2 interacted with bbtFADD2 and bbtASCs. The prodomain of bbtCaspase-1/2 (C2-2) interacted with DD of bbtFADD2 and DED of bbtASCs directly, but prodomains of bbtCaspase-8 (C8-1) and bbtCaspase3-like (C3-1) did not. WB: antibody used in Western blotting analysis. Data show a representative result from at least three separate experiments.

Although phylogenetic analysis clearly showed the evolutionary relationship between *bbtCaspase-1/2 *and vertebrate caspase-1 and caspase-2, the domain architecture of *bbtCaspase-1/2 *is novel. The N-terminal CARD of caspase-1 and caspase-2 in both invertebrates and vertebrates was replaced with a DED and DD in *bbtCaspase-1/2 *(Figure 4A). Thus, to compare its function in amphioxus to that in vertebrates, subcellular localization analysis was conducted. Results showed the fusion protein *bbtCaspase-1/2*-GFP and the truncated mutants containing the DD (C2-2, C2-3, and C2-5) located in the nucleus as dot-like or filamentous structures (Figure 4B), suggesting that the DD is essential for nuclear localization, as is the CARD in human caspase-2 [26]. In spite of the co-localization of *bbtCaspase-1/2 *and amphioxus CASP2 and RIP1 domain containing adaptor with DD (*bbtCRADD*) (see Additional file 4), caspase-9 activity and apoptosis were not observed in *bbtCaspase-1/2 *transfected HeLa cells [27]. However the co-expression of *bbtCaspase-1/2 *and *bbtFADD2 *leads to DD-dependent co-localization of the two proteins as dot-like subcellular structures in the nucleus (Figure 4C). Sequence comparison showed a close identity between the DDs of *bbtCaspase-1/2 *and *bbtFADD2 *(see Additional file 5), indicating that these molecules may be involved in the same signaling pathway. Further co-immunoprecipitation tests confirmed their interaction, dependent on DDs (Figure 5C).

Although IL-1 proteins and ICE-like caspases are absent in the amphioxus genome [20], we found two homologs of mammalian ASC. Both short bbtASC (*bbtASC-S*) and long bbtASC (*bbtASC-L*) contain an N-terminal DED that is a substitute for a PYD in human ASC (Figure 5A) [28]. When *bbtCaspase-1/2 *was co-transfected in HeLa cells with *bbtASC*s, the *bbtCaspase-1/2 *translocated from nucleus to cytoplasm and co-localized with *bbtASC*s (Figure 5B,Additional files 6 and 7) [29,30]. The results of co-immunoprecipitation suggested that *bbtCaspase-1/2 *interacted with *bbtASC*s through the DED (Figure 5C and Additional file 2). Thus, we not only showed the novel domain architecture of *bbtCaspase-1/2*, but also demonstrated that it may be involved in apoptosis through *bbtFADD2*, whose apoptosis function has been characterized previously [22]. In addition, we reported the formation of an inflammasome-like complex through the interaction of *bbtCaspase-1/2 *and *bbtASC *for the first time in this basal chordate.

### The inhibitors of the extrinsic apoptotic pathway-related caspases block amphioxus embryogenesis in early stages

**Figure 6:**
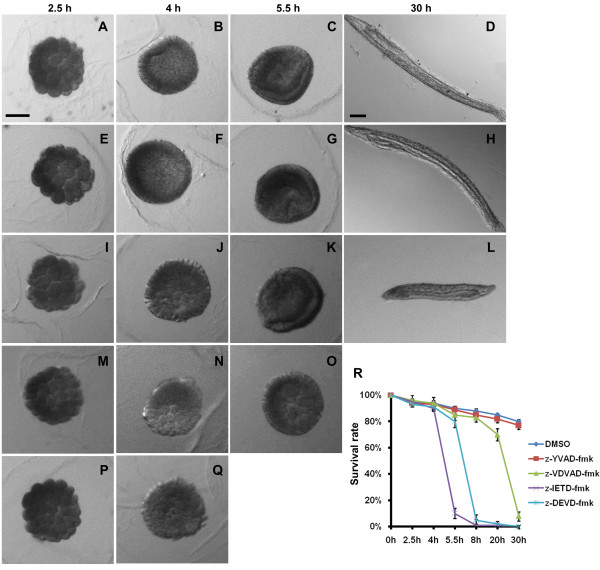
**The effects of four caspase inhibitors on amphioxus embryogenesis**. **(A-D) **Embryos treated with 50 μM DMSO were presented as the negative control. **(E-H) **Embryos were treated with 50 μM caspase-1 inhibitor z-YVAD-fmk and collected at 2.5, 5.5, 8 and 30 h post-fertilization. **(I-L) **Embryos were treated with 50 μM caspase-2 inhibitor z-VDVAD-fmk and collected at 2.5, 5.5, 8 and 30 h post-fertilization. **(M-O) **Embryos were treated with 50 μM effector caspase inhibitor z-DEVD-fmk in which more than 90% of embryos stopped developing at early blastula. **(P-Q) **Embryos were treated with 50 μM caspase-8 inhibitor z-IETD-fmk and more than 90% of embryos stopped developing at late morula. Scale bar, 100 μm. **(R) **The survival rate of each group was calculated with the number of observable eumorphism embryos divided by the number of zygotes at 0, 2.5, 4, 5.5, 8, 20 and 30 h post-fertilization using a somatotype microscope. Results were confirmed by at least three separate experiments. Values were considered to be significant when *P *< 0.05.

Apoptosis is an essential event for development and normal metamorphosis in the metazoan [31]. However, most caspases display low levels in the embryo and little is known of their function in embryogenesis [32]. Immediately following fertilization, we divided embryos into groups treated with four specific caspase inhibitors in dimethyl sulfoxide (DMSO) and a group treated with DMSO alone as a control. The embryos exposed to either DMSO or caspase-1 inhibitor z-YVAD-fmk developed normally. At 30 h, the embryos treated with the caspase-2 inhibitor z-VDVAD-fmk were shorter compared to controls, and showed delayed mouth opening. The other two inhibitors were associated with blocked development at early stages. The effector caspase inhibitor z-DEVD-fmk stopped embryo development at the blastula and the caspase-8 inhibitor z-IETD-fmk blocked embryonic development at the morula (Figure 6).

## Discussion

### The novel domain architecture of the expanded caspase family may have led to the emergence of new apoptotic pathways in amphioxus

Genomic annotations of several lower metazoans have revealed an expansion of the caspase family, including 17 homologous genes in *Hydra *[33], 31 in the sea urchin [32] and 45 in amphioxus [20], compared to only four in the nematode, eight in the fly and twelve in the human. Moreover, compared to those in vertebrates, the death-fold domain (DFD) -containing caspases in amphioxus are expanded by seven- to ten-fold, and include many with novel domain architectures. For example, the single CARD in mammalian caspase-2 is replaced by a tandem DED and DD in *bbtCaspase-1/2*. Although the *bbtCaspase3*-like caspase domain is similar to human caspase-3/7, it unexpectedly contains an N-terminal DED. Since all DFDs function as protein-protein interaction facilitators [34], the domain duplication and shuffling by recombination are probably the main force behind increased or diversified protein functions [35]. Thus, the combining and reshuffling of this domain type in the caspase family provide indications of a greater diversified apoptotic network in amphioxus. This is reflected by the fact that, unlike the reported apoptosis associated with bfCASP-3/7, which has similar domain architecture to human caspase-3/7 [36], the apoptosis mediated by *bbtCaspase3*-like is not completely inhibited by the inhibitor z-DEVD-fmk. In addition, *bbtCaspase3*-like localized in the nucleus independent of its DED, indicating that a previously unreported nuclear cell death pathway may exist in amphioxus, which is distinct from the known caspase-dependent apoptosis.

### The conservation of function of *bbtCaspase-8 *in the extrinsic apoptotic pathway

Although organisms may have evolved distinct apoptosis networks, some core aspects always remained. The extrinsic apoptotic signaling pathway is well known, and was thought to be unique to vertebrates before the publication of sea urchin and amphioxus genomes. Recently, based on ESTs and the whole genome assembly of the ancient metazoan phylum Cnidaria, a DED-containing caspase, a DED-containing FADD-like adaptor protein and a potential death receptor were identified in *Hydra *[33]. In *Drosophila *a death adapter, dFADD, which is homologous to the mammalian FADD, was found to bind death-related CED-3-like protein (DREDD) through the death inducing domain (DID), a novel DFD involved in caspase adapter interactions and promoting cell death activity [25]. Our study identified an ortholog of mammalian caspase-8 in Chinese amphioxus, *bbtCaspase-8*. This gene contains two tandem DEDs, as does its mammalian counterpart, and also interacts with *bbtFADD1 *through the DEDs to induce mammalian cell death. These results suggest that the core molecules participating in the extrinsic pathway arose early, before the vertebrate, and were conserved in Eumetazoa. In HeLa cells, *bbtFADD1 *overexpression alone was not found to induce apoptosis [22], and co-localized with the prodomain of *bbtCaspase-8 *only, indicating that FADD and the initiator caspase evolved dependently. The FADD proteins are likely specific to cognate initiator caspases, but the proteolytic cascade processes of caspases are generally conserved among species.

### The association of *bbtCaspase-1/2 *with *bbtASC*s sets up the foundation for vertebrate pro-inflammatory caspases in regulating inflammation

In vertebrates, NLRs usually recruit the adaptor ASC via PYD-PYD interaction. Subsequently, the N-terminal CARD within ASC recruits caspase-1 to form a complex known as an inflammasome [11]. Although five ICE-like genes have been found in the sea urchin genome, none are linked to the CARD prodomain, as is the case in vertebrates, and pro-inflammatory functions of caspases have not been identified in sea urchins. Here, *bbtCaspase-1/2 *with the highest similarity to mammalian caspase-2 and caspase-1 and two ASC molecules with novel domain architectures were identified in amphioxus. In addition, we demonstrated that *bbtASC*s interacted with *bbtCaspase-1/2 *and led *bbtCaspase-1/2 *to translocate from the nucleus to the cytoplasm. However, no effects on the maturation of IL-1β and IL-18 were observed in different mammalian cells transfected with both *bbtCaspase-1/2 *and *bbtASC*s by ELISA testing. One of the possibilities is that the functions of *bbtCaspase-1/2 *and *bbtASC*s could not be represented in mammalian cell lines, since the PYD in mammalian ASC was replaced with a DED both in *bbtASC-L *and *bbtASC-S *and a DED and DD tandem prodomain architecture in *bbtCaspase-1/2 *exists only in amphioxus. To date, there are no homologs of human IL-1 and IL-18, which are important products of mammal inflammasomes, found in the amphioxus genome. Moreover, from the analysis of the amphioxus genome, the PYDs of amphioxus NLRs were also replaced by DEDs. Thus, although the interacting basic for the inflammasome-like complex has been established, the mechanism and the effectors in amphioxus should be completely different from those in mammals given their different structures. Here, we also found that *bbtCaspase-1/2 *co-localizes with *bbtFADD2 *in the nucleus through the DD, but not with *bbtCRADD *[26]. In addition, we previously identified that translocation of *bbtFADD2 *from the nucleus to the cytoplasm induces apoptosis in HeLa cells [22]. Although the underlying mechanism of *bbtFADD2 *and *bbtCaspase-1/2 *in amphioxus apoptosis needs to be tested in future successful cultured amphioxus cells, our results still implied that *bbtCaspase-1/2 *may be involved in an unidentified apoptotic pathway in the nucleus. Thus, *bbtCaspase-1/2*, which arose from domain recombination, may have emerged as a molecular switch in controlling the balance between caspase-dependent innate immune response and apoptosis in the basal chordate.

### Effect of caspase related proteins on amphioxus early embryogenesis

The cell death pathways were originally found in the study of *C. elegans *and *Drosophila *development and are essential for normal development of body plan and organ structures. Some knockout gene mouse studies have been conducted in which caspase-1 deficient mice showed no apparent gross abnormalities [37]; caspase-2 deficient mice were devoid of severe phenotypic abnormalities, with caspase-2 having an impact on neuron apoptosis [38]; caspase-3 and -7 deficient mice died immediately after birth [39]; and caspase-8 deficient mouse fetuses did not survive past mid-gestation [40]. As for amphioxus, apoptosis has been examined during amphioxus development and one related protein, bfCASP-3/7 has been proved to be a substrate of caspase inhibitor z-DEVD-fmk *in vitro *[36]. Apart from the bfCASP-3/7 and the three caspase genes we have identified here, there are at least 40 other caspase related genes. How such expanded caspase related proteins, including the proteins with caspase-like proteolytic activity, like metacaspases [41,42], are involved in amphioxus development is not known. To address this question, we used several specific caspase inhibitors to treat amphioxus embryos. The embryos treated with caspase-1 inhibitor developed normally. It is possible that no homolog of mammalian caspase-1 exists in the amphioxus genome, so its inhibitor has no target in amphioxus. Alternatively, this protein may have its expression and function in the adult phase, associated with the immune system, because caspase-1 is well-known as an inflammatory caspase in vertebrates. In the sea urchin, ICE-like caspase expression has not been detected in either embryo or larva [32], and our RT-PCR results indicate that the expression of *bbtCaspase-1/2 *is much higher than the other two analyzed in adult tissues. Thus, although *bbtCaspase-1/2 *may interact with *bbtFADD2*, this potential apoptotic pathway most likely participates in the development of gut openings (mouth and gill slits) and body growth in larvae, but does not influence early embryogenesis, as was seen in the group treated with the caspase-2 inhibitor. Caspase has been reported to show earliest expression in the mesoderm of the gastrula [43], and bfCASP-3/7 is expressed from gastrula to larva [36]. Overexpression of bfCASP-3/7 induced mammalian cell apoptosis and the inhibitor z-DEVD-fmk blocked its activity [36]. All of these evidences suggested that bfCASP-3/7 is likely to be one of the effector caspase related proteins inhibited by z-DEVD-fmk in embryogenesis. Moreover, *bbtCaspase-8 *induced HeLa cell apoptosis and such activity can be inhibited by z-IETD-fmk, indicating that z-IETD-fmk may inhibit the activities of caspase-8 related proteins during embryogenesis. In addition, we found that initiator caspases-related proteins may function before effector caspases. Thus, these caspases mediate extrinsic apoptosis, which is indispensable in early embryogenesis, probably by inducing cell differentiation and embryonic layer formation.

## Conclusions

Our studies not only indicated that the function of DR-mediated extrinsic apoptosis is conserved and completely formed in amphioxus, but also provided the first evidence of the interacting foundation for the inflammasome-like complex in a non-vertebrate. An interesting point is that many amphioxus caspase genes possess novel domain architectures, which provides important insights into understanding how new signaling pathways emerged along with domain recombination and shuffling, especially at the transition from invertebrates to vertebrates.

## Methods

### Animals and embryos

Adult Chinese amphioxus (*Branchiostoma belcheri tsingtauense*) were obtained from Kioachow Bay near Qingdao, China. During May and June, ripe males and females, after a 14:10 h light:dark cycle at 22°C for at least seven days, were induced to spawn by thermal shock at 26°C for 36 h. Subsequently, the animals were placed individually in plastic bottles with a small amount of filtered seawater and held in dark. Spawning was checked every hour using red light and the eggs and sperm collected. After *in vitro *fertilization, isogenous embryos were divided into five groups, four treated with a different caspase inhibitor (Merck, NJ, USA) at 50 μM in DMSO and one with DMSO alone. The embryos were allowed to develop to the desired stage at 23°C.

### Sequence retrieval, structural and phylogenetic analyses

The protein sequences of the caspase family were used to perform BLASTp searches against all databases available at the National Center for Biotechnology Information and the DOE Joint Genome Institute. Domain searches were performed against the PROSITE. Protein sequences were first aligned using ClustalX 1.83 and manually corrected using GeneDoc. Then, the neighbor joining tree was obtained using the routine in MEGA 3.1 with 1000 bootstrap tests.

### Cloning of *bbtCaspase-8, bbtCaspase-1/2*, and *bbtCaspase3*-like cDNAs

Full-length cDNA sequences of three genes were identified in the total cDNA library of Chinese amphioxus. For each, a partial sequence was cloned from Chinese amphioxus cDNA using a specific primer pair derived from *B. floridae*. Subsequently, 5'-rapid amplification of cDNA ends (RACE) and 3'-RACE were performed according to the manufacturer's protocol of the GeneRacer kit (Invitrogen, USA). After sequencing and manual alignment, we obtained the full-length sequences of *bbtCaspase-8, bbtCaspase-1/2*, and *bbtCaspase3*-like. Then, gene specific primers were designed and full-length cDNA sequences of the three amphioxus caspases were obtained from the cDNA library of adult Chinese amphioxus and inserted into T easy vectors for following vectors constructions. Human caspases were cloned following similar procedures with the primers derived from GenBank. *BbtCaspase-8, bbtCaspase-1/2 *and *bbtCaspase3*-like sequences have been deposited in the GenBank database [GenBank:JF717867]; [GenBank: JF717868]; [GenBank:JF717869].

### Cell lines and expression plasmids

HeLa cells were maintained in DMEM supplemented with 10% fetal calf serum (GIBCO, USA). The ORF and PCR fragments encoding for amino acids 1-193 and 194-606 of *bbtCaspase-8*; 1-90, 1-226, 91-226, 227-544, 91-544 and 1-90aa+227-544aa of *bbtCaspase-1/2*; 1-100 and 101-446 of *bbtCaspase3*-like were cloned by PCR and inserted into vectors pEGFP-N1 and pCMV (Clontech, USA) with a N-terminal HA or FLAG epitope tag, respectively, and designated C8-1, C8-2; C2-1, C2-2, C2-3, C2-4, C2-5, C2-6; C3-1, and C3-2. The ORFs of hsCaspase-3 and hsCaspase-8 were inserted into vectors pEGFP-N1 and pCMV respectively.

### Real-time PCR

Either 10^5 ^colony forming units (CFU) of *S. aureus *or *V. vulnificus *in PBS were injected into the amphioxus celom and cultured in separate tanks. The challenged animals were collected at 2, 4, 8, 12, 24, 36, 48 and 72 h post-injection. The protocol for the control animals injected with PBS only followed the same schedule. Intestine from five individuals were combined in a single sample for total RNA extraction and subjected to reverse transcription (TOYOBO, Japan). Total RNA from various tissues of un-challenged Chinese amphioxus were extracted and treated with the same way. The 208 bp product of bbtCaspase-8 was amplified by primer pair: 5'-GTCATCGTCAACAACAAAC-3' and 5'-TGGAGTGGTCTTCATAGC-3'; the 113 bp product of bbtCaspase-1/2 was amplified by primer pair: 5'-TTAAGAGCGAGATGAGAAG-3' and 5'-TAGTTGTGTTGCGTATCC-3'; the 186 bp product of bbtCapase3-like was amplified by primer pair: 5'-GGAGATGGAACAGGATGAG-3' and 5'-GAAGACGAGGACGATTGG-3'. All samples were analyzed by RT-PCR in triplicate under the following conditions: 2 min at 95°C followed by 40 cycles of 15 s at 95°C, 15 s at 60°C, and 20 s at 72°C. Data were quantified with the 2^-ΔΔCt ^method based on Ct values of *bbtCaspase-8, bbtCaspase-1/2, bbtCaspase3*-like and β-actin from two parallel experiments done in triplicate. For expression following challenge, folds were normalized to the expression in PBS-injected animals. Values were considered to be significant at *P *< 0.05.

### Immunofluorescence imaging

HeLa cells were seeded on coverslips (10 mm × 10 mm) in a 24-well plate for more than 12 h and transfected with the indicated expression plasmids using jetPEI (Polyplus Transfection, France) according to the manufacturer's instructions. Twenty to twenty-four hours after transfection, cells were fixed in 4% formaldehyde solution for 15 min. Coverslips were washed with PBST (0.05% Tween-20 in PBS) three times and blocked with 5% BSA in PBST at room temperature for one hour. Primary monoclonal antibodies (Sigma, USA) were added at 1 mg/mL in blocking buffer for 1 hr, and secondary antibodies (1:5000) (Invitrogen, USA) were added for 1 h with three washes between each step. Samples were finally stained with 0.2 μg/mL 4',6-diamidino-2-phenylindole (DAPI) in PBS for 5 min, washed three times in PBS and mounted in MOWIOL R4-88 Reagent (Calbiochem, USA). Fluorescence images were photographed with a Zeiss AxioVision 4 microscope (63 × objective) with appropriate filters.

### Annexin V binding apoptosis assay

HeLa cells were cultured in 12-well plates and transfected with 2 μg fused plasmids in pCMV vector for each well. At 36 h post-transfection, cells were trypsinized and collected for analysis with the PE Annexin V Apoptosis Detection Kit I (BD Pharmingen, USA) according to the manufacturer's instructions. The samples were analyzed by a BD FACSCaliburTM cytometer (Becton Dickinson, Heidelberg, Germany). For each sample, 10,000 gated events were required. Annexin V positive cells were considered apoptotic, and the sample transfected empty pCMV vectors were used as a negative control. The positive control was treated with camptothecin (Sigma) at 10 μM for 24 h. Camptothecin was freshly dissolved in DMSO at 10 mM and further diluted in DMEM before each experiment.

### Caspase activity assay

HeLa cells were cultured in 48-well plates and transfected with 400 ng/well of indicated plasmids. At 20 h post-transfection, the caspase activity of all samples was measured by Caspase-Glo^® ^3/7 Assay or Caspase-Glo^® ^8 Assay (Promega, USA) according to the manufacturer's instructions. The samples transfected empty vectors were used as negative controls and transfected human caspases were positive controls.

### Co-immunoprecipitation

HeLa cells in 6-well dishes were transfected with 4 μg of indicated plasmids in each well (2 μg/each expression vector). More than 24 h after transfection, the whole cell extracts were prepared in immunoprecipitation lysis buffer [50 mM Tris, pH 7.4, 150 Mm NaCl, 1% Nonidet P-40, 0.5% deoxycholic acid sodium salt, and cocktail protease inhibitor (Roche, Germany)] and incubated with primary antibodies at 4°C overnight, then incubated with Protein G Sepharose (Roche) at 4°C for 4-6 h. The mix was washed three times with lysis buffer. Analysis was conducted using SDS-PAGE followed by Western blot with enhanced chemiluminescent reagent (Amersham Pharmacia, Finland). The monoclonal antibody against HA epitope tag (1:5000), FLAG epitope tag (1:1000), and the anti-mouse secondary antibody (1:5000) were purchased from Sigma.

## Abbreviations

ASC: apoptosis associated speck-like protein containing a CARD; bbt: *Branchiostoma belcheri tsingtauense*; BLASTp: protein-protein basic local alignment search tool; bp: base pair; BSA: bovine serum albumin; CARD: caspase recruitment domain; CRADD: CASP2 and RIP1 domain containing adaptor with death domain; DD: death domain; DED: death effector domain; DFD: death-fold domain; DMSO: dimethyl sulfoxide; DMEM: Dulbecco's modified Eagle's medium; DR: death receptor; DREDD: death-related CED-3-like protein; ELISA: enzyme-linked immunosorbent assay; EST: expressed sequence tag; FADD: Fas-associated death domain; GFP: green fluorescent protein; IL: interleukin; NLR: NOD-like receptor; ORF: open reading frame; PBS: phosphate buffered saline; PYD: pyrin domain; RACE: rapid amplification of cDNA ends; RT-PCR: real time polymerase chain reaction; TNFR: tumor necrosis factor receptor; z-IETD-fmk: benzyloxycarbonyl-Ile-Glu(OMe)-Thr-Asp(OMe)-fluoromethylketone; z-DEVD-fmk: benzyloxycarbonyl-Asp(OMe)-Glu(OMe)-Val-Asp(OMe)-fluoromethylketone; z-VDVAD-fmk: benzyloxycarbonyl-Val-Asp(OMe)-Val-Ala-Asp(OMe)-fluoromethylketone; z-YVAD-fmk benzyloxycarbonyl-Tyr-Val-Ala-Asp(OMe)-fluoromethylketone.

## Competing interests

The authors declare that they have no competing interests.

## Authors' contributions

LQX designed the research, performed the experiments and wrote the paper. SCY and ALX designed the research, analyzed the data and revised the paper. JL, JR, MYY and HQH performed part of the experiments. SFH, SWC and ZHR analyzed the data. All authors read and approved the final manuscript.

## Supplementary Material

Additional file 1 **Alignment of caspase domain sequences among bbtCaspase3-like, amphiCASP-3/7, human caspase-3 and caspase-7**. Click here for file

Additional file 2 **Alignment of DED sequences among bbtCaspases, bbtFADDs and bbtASCs**. Click here for file

Additional file 3 **Alignment of caspase domain sequences between bbtCaspase-8 and hsCaspase-8**. Click here for file

Additional file 4 **BbtCaspase-1/2 did not co-localize with bbtCRADD in HeLa cells**. Click here for file

Additional file 5 **Alignment of DD sequences among bbtCaspase-1/2, bbtFADD1 and bbtFADD2**. Click here for file

Additional file 6 **Time-lapse video of overlay fluorescences of a HeLa cell co-transfected with bbtCaspase-1/2-GFP and bbtASC-RFP**. The video was filmed using a Zeiss microscope for 2 h, and it started filming at 6 h after transfection. Click here for file

Additional file 7 **The same video showing only the green channel, bbtCaspase-1/2-GFP**. Click here for file
