# Supplementary material for: The conservation and uniqueness of the caspase family in the basal chordate, amphioxus
Source: BMC Biol. 2011 Sep 21;9:60. doi: 10.1186/1741-7007-9-60 (PMC3196919; doi:10.1186/1741-7007-9-60)
Supplement: Additional file 1 — Alignment of caspase domain sequences among bbtCaspase3-like, amphiCASP-3/7, human caspase-3 and caspase-7. [file 1741-7007-9-60-S1.DOC]

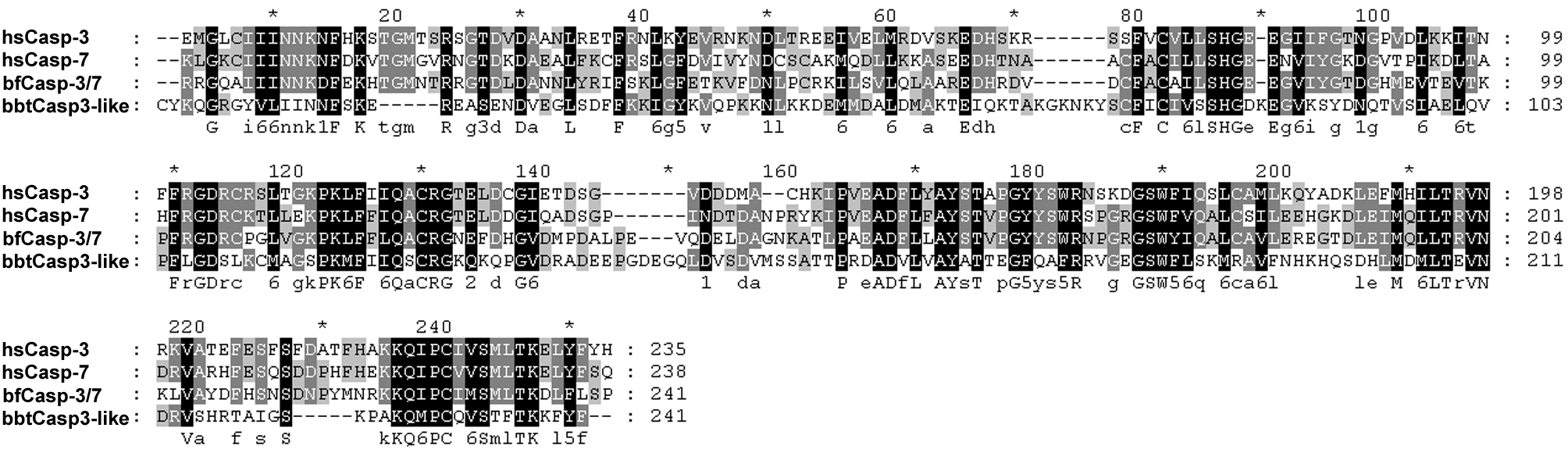


**Figure S1.** Alignment of caspase domain sequences among bbtCaspase3-like, amphiCASP-3/7, human caspase-3 and caspase-7. Black and gray shading indicate ≥80% amino acid sequence identity and similarity.
