# Supplementary material for: The conservation and uniqueness of the caspase family in the basal chordate, amphioxus
Source: BMC Biol. 2011 Sep 21;9:60. doi: 10.1186/1741-7007-9-60 (PMC3196919; doi:10.1186/1741-7007-9-60)
Supplement: Additional file 2 — Alignment of DED sequences among bbtCaspases, bbtFADDs and bbtASCs. [file 1741-7007-9-60-S2.DOC]

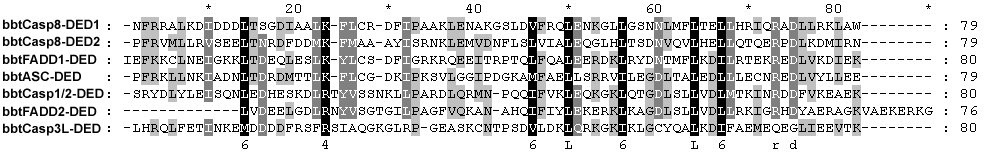


**Figure S2.** Alignment of DED sequences among bbtCaspases, bbtFADDs and bbtASCs. These indicated that the DED of bbtCaspase-8 is similar with that of bbtFADD1 and the DED of bbtCaspase-1/2 is similar with that of bbtASCs. Black and gray shading indicate ≥80% amino acid sequence identity and similarity.
