# Supplementary material for: The conservation and uniqueness of the caspase family in the basal chordate, amphioxus
Source: BMC Biol. 2011 Sep 21;9:60. doi: 10.1186/1741-7007-9-60 (PMC3196919; doi:10.1186/1741-7007-9-60)
Supplement: Additional file 3 — Alignment of caspase domain sequences between bbtCaspase-8 and hsCaspase-8. [file 1741-7007-9-60-S3.DOC]

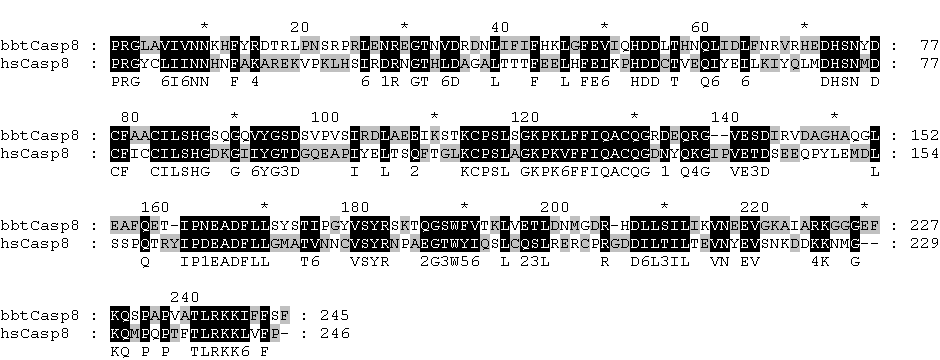


**Figure S3.** Alignment of caspase domain sequences between bbtCaspase-8 and human caspase-8. These indicated that the catalytic sequences of bbtCaspase-8 is conservative with that of hsCaspase-8. Black and gray shading indicate ≥80% amino acid sequence identity and similarity.
