# Supplementary figures and images for: The conservation and uniqueness of the caspase family in the basal chordate, amphioxus
Source: BMC Biol. 2011 Sep 21;9:60. doi: 10.1186/1741-7007-9-60 (PMC3196919; doi:10.1186/1741-7007-9-60)

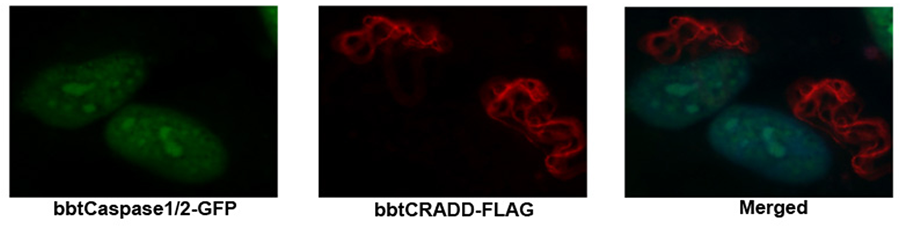


**Figure S3.** BbtCaspase-1/2 did not co-localize with bbtCRADD in HeLa cells.

Supplement: Additional file 4 — BbtCaspase-1/2 did not co-localize with bbtCRADD in HeLa cells. [file 1741-7007-9-60-S4.DOC]
