# Supplementary material for: The conservation and uniqueness of the caspase family in the basal chordate, amphioxus
Source: BMC Biol. 2011 Sep 21;9:60. doi: 10.1186/1741-7007-9-60 (PMC3196919; doi:10.1186/1741-7007-9-60)
Supplement: Additional file 5 — Alignment of DD sequences among bbtCaspase-1/2, bbtFADD1 and bbtFADD2. [file 1741-7007-9-60-S5.DOC]

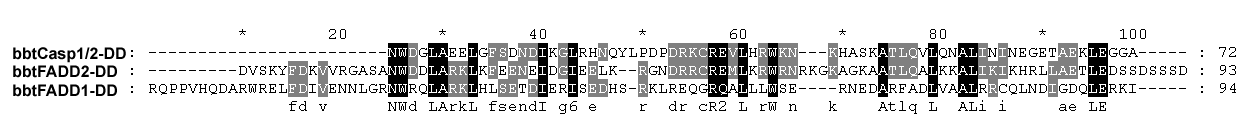


**Figure S4.** Alignment of death domain sequences among bbtCaspase-1/2, bbtFADD1 and bbtFADD2. The DD sequences of bbtCaspase1/2 is more similar with bbtFADD2 than bbtFADD1. Black and gray shading indicate ≥80% amino acid sequence identity and similarity.
